# Purification of a specific native genomic locus for proteomic analysis

**DOI:** 10.1093/nar/gkt822

**Published:** 2013-09-11

**Authors:** Stephanie D. Byrum, Sean D. Taverna, Alan J. Tackett

**Affiliations:** ^1^Department of Biochemistry and Molecular Biology, University of Arkansas for Medical Sciences, Little Rock, AR 72205, USA and ^2^Department of Pharmacology and Molecular Sciences, Johns Hopkins School of Medicine, Baltimore, MD 21205, USA

## Abstract

Here, we describe an approach to isolate native chromatin sections without genomic engineering for label-free proteomic identification of associated proteins and histone post-translational modifications. A transcription activator-like (TAL) protein A fusion protein was designed to recognize a unique site in the yeast *GAL1* promoter. The TAL-PrA fusion enabled chromatin affinity purification (ChAP) of a small section of native chromatin upstream from the *GAL1* locus, permitting mass spectrometric (MS) identification of proteins and histone post-translational modifications regulating galactose-induced transcription. This TAL-ChAP-MS approach allows the biochemical isolation of a specific native genomic locus for proteomic studies and will provide for unprecedented objective insight into protein and epigenetic mechanisms regulating site-specific chromosome metabolism.

## INTRODUCTION

One of the most compositionally diverse structures in a eukaryotic cell is a chromosome. A multitude of macromolecular protein interactions and epigenetic modifications must properly occur on chromatin to drive functional aspects of chromosome biology like gene transcription, DNA replication, recombination, repair and sister chromatid segregation. Analyzing how proteins interact *in vivo* with chromatin to direct these activities and how epigenetics factors into these mechanisms remains a significant challenge owing to the lack of technologies to comprehensively analyze protein associations and epigenetics at specific native chromosome sites. Chromatin immunoprecipitation (ChIP) assays have traditionally been used to better understand genome-wide distributions of chromatin-associated proteins and histone post-translational modifications (PTMs) at the nucleosome level (1–7). However, major drawbacks of current ChIP-based methods include their confinement to examining singular histone PTMs or proteins rather than simultaneous profiling of multiple targets, the inability of ChIP to directly determine the co-occupancy of particular histone PTMs and that ChIP is reliant on the previous identification and development of affinity reagents against the molecular target. A more comprehensive and unbiased approach would be the biochemical isolation of a specific native genomic locus for proteomic identification of proteins-associated and histone PTMs. Similar approaches have been performed for large structures like telomeres, engineered plasmids or engineered loci (8–13); however, the proteomic analysis of a small native genomic region without genomic engineering for specifically associated proteins and histone PTMs has yet to be demonstrated. To work toward proteomic studies of native chromatin regions (i.e. sections of chromatin that are unaltered genetically and spatially the genome), we recently developed a technique termed Chromatin Affinity Purification with Mass Spectrometry (ChAP-MS) that provides for the enrichment of a native 1-kb section of a chromosome for site-specific identification of protein interactions and associated histone PTMs (13). This ChAP-MS approach uses the association of an ectopically expressed affinity-tagged LexA protein with a genomically incorporated LexA DNA binding site for site-specific chromatin enrichment. The ChAP-MS approach provides for the isolation of chromatin from the native site in the chromosome; however, one must genomically engineer a LexA DNA binding site, which could alter the native state of the chromatin and which requires a biological system readily amendable to genomic engineering.

**Figure 1.**
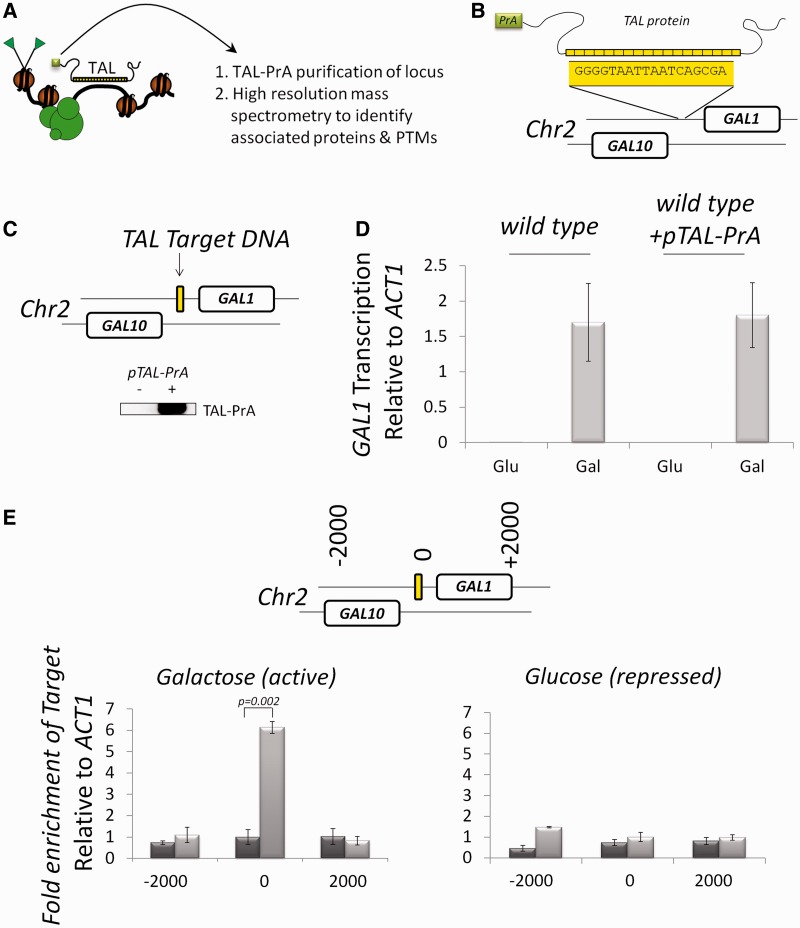
TAL proteins can specifically enrich native chromatin sections. (**A**) Schematic overview of TAL-ChAP-MS technology. (**B**) A unique DNA sequence in the promoter region of *GAL1* was used to design a specific binding TAL protein that contained a PrA affinity tag. (**C**) A *pTAL-PrA* plasmid was introduced into *S. cerevisiae* cells, and the constitutive expression of the TAL-PrA fusion protein was confirmed by western blotting for PrA. (**D**) Expression of TAL-PrA does not impede galactose-induced *GAL1* transcription. cDNA from wild-type yeast and wild-type with a plasmid expressing PrA-tagged TAL (*+pTAL-PrA*) grown in glucose (Glu) or galactose (Gal) was used as a template for real time PCR analysis of *GAL1* versus *ACT1* gene transcription. Error bars are the standard deviation. (**E**) TAL-PrA specifically binds and enriches chromatin at the promoter of transcriptionally active *GAL1*. ChIP was performed to the PrA-tag in wild-types cells containing the TAL-PrA (+*pTAL-PrA*, light gray bars) and in wild-type control (dark gray bars). The efficiency of *GAL1* enrichment relative to *ACT1* was monitored by real-time PCR with primers targeted to the TAL binding site (‘0’) and to DNA sequences 2000 bp up- and downstream. The standard deviation is indicated.

To alleviate genomic engineering for affinity enrichment of chromatin sections, we report the use of modified transcription activator-like (TAL) effector proteins to site-specifically target a native section of a chromosome for purification and proteomic analysis. We term this approach TAL-ChAP-MS (Figure 1A). TAL effector proteins are from *Xanthomonas*, which infects plants and translocates TAL effectors into cells where they serve as transcription activators (14–16). TALs contain a central domain of 18 tandem repeats of 34 amino acids each, which direct sequence-specific DNA binding (16,17). Binding to a given nucleobase in DNA is determined by two adjacent amino acids (12 and 13) within each of the 18 repeats (14). Thus, by mutating these amino acids in each of the 18 tandem repeats, one can ‘program’ binding to a given 18-nt region of DNA *in vivo*. TAL proteins have been validated in cell culture for targeting nucleases for genome editing and for targeting transcription activators (18,19). To test the ability of a TAL protein to serve as an affinity enrichment reagent for native chromatin isolation, a TAL protein was designed that bound a unique 18-nt region of DNA in the promoter region of the *GAL1* gene in *Saccharomyces cerevisiae* (Figure 1B). We chose to analyze proteins and histone PTMs regulating the galactose-induced gene transcription of *GAL1* because (1) this is a well-studied genomic locus, which will provide for proof-of-principle analysis, and (2) we previously used this locus to develop the ChAP-MS technique (13); thus, a comparison can be made to the TAL-ChAP-MS approach.

One of the major complications for studying specific protein associations with purified protein complexes or with chromatin is the co-enrichment of non-specifically associating proteins. This particularly becomes an issue when studying low copy number entities such as a single genomic locus. With the advancement of high-resolution and sensitive mass spectrometry in recent years, it has been suggested that >10^9 cell equivalents are needed to study single genomic loci with proteomic approaches (20). In agreement, our ChAP-MS studies used 10^11 cells for isolation of *GAL1* promoter chromatin at levels sufficient for proteomic analysis (13). When scaling up purifications of low copy entities to meet the sensitivity necessary for high-resolution mass spectrometric analysis, the issue of co-purifying abundant non-specific proteins becomes a major challenge. In the ChAP-MS approach (13), we used an isotope-labeling strategy to categorize whether a protein co-enriching with a section of chromatin was specifically associated or a contaminant. Limitations for isotope-labeling approaches are cost and having biological systems of study that are amendable to stable isotope-labeling with amino acids. To circumvent the use of isotope-labeling, we now have incorporated label-free quantitative mass spectrometry in the TAL-ChAP-MS workflow. The described TAL-ChAP-MS approach can therefore provide for the purification of a native chromatin region for label-free quantitative proteomic analysis, which will greatly simplify studies of how proteins and combinatorial histone PTMs regulate chromosome metabolism.

## MATERIALS AND METHODS

### pTAL-PrA plasmid, real-time rtPCR and ChIP

For affinity enrichment of chromatin from the promoter region of the *GAL1* gene in *S. cerevisiae*, a TAL protein was designed (by the GeneArt Precision TAL services of Life Technologies) to bind a unique 18-nt sequence (GGGGTAATTAATCAGCGA) 193 base pairs upstream of the *GAL1* open-reading frame (Figure 1B). The TAL protein was designed as a truncation that lacked the native N-terminal transcription activation domain, but it contained the site-specific DNA-binding region. To develop an affinity enrichment reagent, the LexA-coding region of *pLexA-PrA* [plasmid that constitutively expresses a PrA-tagged LexA protein under *TRP* selection; from (13)] was replaced with the TAL-coding region to generate *pTAL-PrA*. Real-time quantitative PCR measurement of galactose-induced transcription of *GAL1*, and all ChIP studies were performed as reported in (13).

### TAL-ChAP-MS

**Figure 2.**
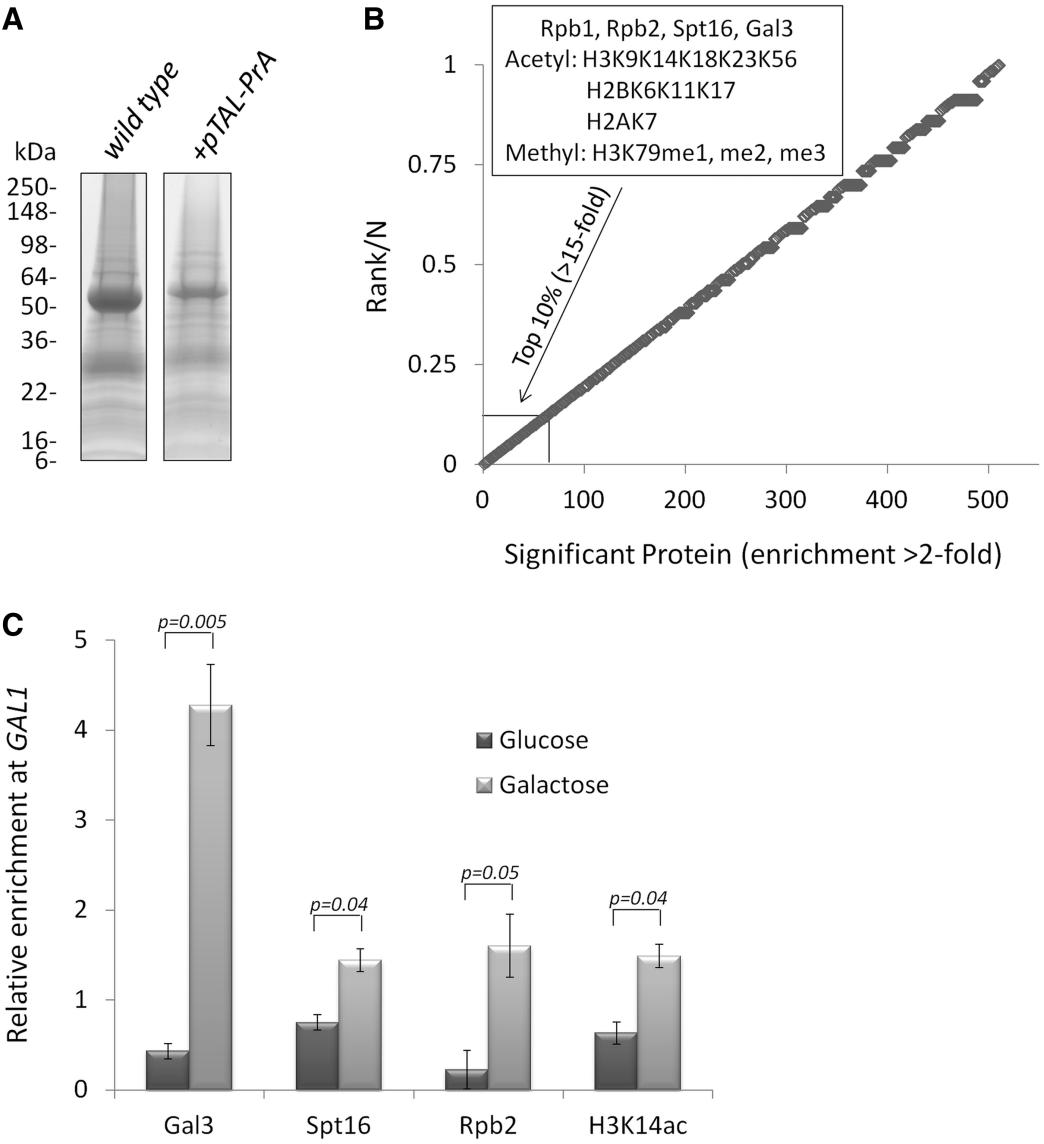
TAL-ChAP-MS analysis of *GAL1* promoter chromatin from cells grown in galactose. (**A**) Proteins co-purifying with TAL-PrA targeted to the promoter region of *GAL1* (+*pTAL-PrA* lane) and proteins non-specifically associating with the IgG-coated Dynabeads (wild-type lane) were resolved by SDS–PAGE/Coomassie-staining and identified by high-resolution mass spectrometry. (**B**) Proteins found by label-free proteomic analysis to be enriched by >2-fold with transcriptionally active *GAL1* promoter chromatin are plotted in accordance to their ranked level of enrichment divided by the total number of enriched proteins (N). Highlighted are the top 10% of proteins (>15-fold enrichment) and histone PTMs enriched with *GAL1* promoter chromatin. (**C**) ChIP was targeted to Spt16-TAP, Rpb2-TAP, Gal3-TAP and H3K14ac under transcriptionally active galactose (light gray bars) and repressive glucose (dark gray bars) growth conditions. ChIP to general H3 was used as a nucleosome occupancy control for H3K14ac ChIP. Enrichment adjacent to the TAL binding site in the promoter of *GAL1* relative to *ACT1* was monitored by real-time qPCR. The standard error is indicated.

To test the TAL-ChAP-MS approach at the promoter region of *GAL1*, wild-type and wild-type (+*pTAL-PrA*) *S. cerevisiae (W303 matA)* cells were cultured to mid-log phase in 3% galactose-containing media, subjected to 1.25% formaldehyde cross-linking, cryogenically lysed and subjected to sonication to shear genomic DNA to ∼1 kb [as detailed in (13,21,22)]. Immunoglobulin G (IgG)-coated Dynabeads were added to lyste from ∼10^11 cells from each growth separately [as detailed in (13)]. Proteins co-enriching with the TAL-PrA (wild-type cells +*pTAL-PrA* lysate) or proteins non-specifically binding to the Dynabeads (wild-type cell lysate) were resolved by SDS–PAGE/Coomassie-staining (Figure 2A), excised as 2-mm bands from the entire gel lane, digested in-gel with trypsin and subjected to high-resolution tandem mass spectrometric analysis with a Thermo Velos Orbitap mass spectrometer [as reported in (13)]. Proteins and typical histone PTMs (lysine acetylation and methylation) were identified using Mascot (Supplementary Tables S1 and S2). To measure enrichment of a protein, the normalized spectral abundance factor (23) was calculated for each protein in each lane by dividing the number of spectral counts (normalized for the size of the protein) of a given protein by the sum of all normalized spectral counts of all proteins in the gel lane (24). The enrichment level for each protein was identified by calculating the fold enrichment (TAL-PrA/wild type) using the normalized spectral abundance factor values (Supplementary Table S1). Proteins with a fold enrichment >2 (511 of 1459 proteins identified) were used to generate a quantile plot of fold enrichment with *GAL1* promoter chromatin (Figure 2B).

## RESULTS

### Overview

A schematic of the TAL-ChAP-MS approach to purify native chromatin for proteomic analysis is shown in Figure 1A. To demonstrate the utility of the TAL-ChAP-MS approach, we used a TAL protein to target the promoter chromatin region upstream of the galactose-inducible *GAL1* gene in *S. cerevisiae* (Figure 1B). Yeast cells were grown in the presence of galactose to induce transcription of the *GAL1* gene, which will recruit proteins and histone PTMs that activate transcription. A wild-type culture and a culture of cells containing a plasmid that expressed a PrA-tagged TAL protein that bound the *GAL1* promoter region were grown and subjected to *in vivo* formaldehyde cross-linking to preserve chromatin structure during purification (21,22). Following cryogenic cell lysis and sonication of chromatin sections to ∼1 kb, each lysate was independently subjected to affinity enrichment of PrA with IgG-coated Dynabeads. Proteins co-enriching with TAL-PrA from the cells containing the *pTAL-PrA* plasmid and those enriching as contamination from the control cells with no plasmid were identified by high-resolution mass spectrometry. Using label-free quantitative analyses, the relative enrichment of proteins and histone PTMs specifically bound to the *GAL1* promoter chromatin were identified.

### Development of TAL-ChAP-MS

*Saccharomyces cerevisiae* cells were transformed with *pTAL-PrA*, and protein expression was validated by western blotting (Figure 1C). To evaluate whether TAL-PrA expression affected galactose-induced transcription of *GAL1*, cDNA was prepared from wild-type and wild-type (+*pTAL-PrA*) cells under glucose (transcriptionally repressed *GAL1*) and galactose (transcriptionally active *GAL1*) growth conditions. Quantitative PCR of this cDNA revealed that expression of TAL-PrA did not affect galactose-induced *GAL1* transcription (Figure 1D). To determine whether TAL-PrA enriched chromatin at the *GAL1* promoter region, ChIP was performed to the PrA-tag in cells from glucose and galactose growths (Figure 1E). Under transcriptionally active conditions, TAL-PrA specifically enriched chromatin from the *GAL1* promoter region relative to sequences 2 kb up- and downstream. The level of chromatin enrichment by TAL-PrA under transcriptionally active conditions was similar to the level used for proteomic studies with LexA-PrA affinity enrichment in the ChAP-MS approach (13). Interestingly, the TAL-PrA protein did not show enrichment of the *GAL1* promoter chromatin under transcriptionally repressive glucose growth conditions. One possibility of many is that the lack of enrichment could be due to inaccessibility of the TAL-PrA to the genomic target due to altered chromatin structure under transcriptionally repressive conditions—highlighting the importance for measuring specific chromatin enrichment of the TAL protein before using this approach for specific chromatin enrichment. In the previous publication of the ChAP approach (13), a LexA-PrA was targeted just upstream of the start codon of *GAL1* for enrichment of chromatin, which showed enrichment under both glucose and galactose growth conditions. Importantly, the TAL used in the current study was targeted 193 bp upstream of the target site of LexA, which suggests that proximal regions may be differentially accessible to DNA-binding affinity reagents under various transcriptional states. In addition to analyzing enrichment of *GAL1* chromatin relative to proximal sequences (Figure 1E), enrichment of *GAL1* chromatin was measured relative to the five most homologous sequences in the genome (Supplementary Table S3). The *GAL1* target DNA showed 4.6-fold better enrichment relative to the next five most similar sites in the genome—demonstrating specificity of the TAL protein used in this study to the targeted sequence at the *GAL1* promoter region. Collectively, the data in Figure 1C–E and Supplementary Table S3 demonstrate that the TAL-ChAP-MS approach can provide enriched chromatin from the *GAL1* promoter under transcriptionally active conditions that would be suitable for proteomic studies.

### Using TAL-ChAP-MS to identify proteins and histone PTMs at the GAL1 promoter

As detailed in the Materials and Methods section, chromatin from the transcriptionally active *GAL1* promoter was enriched with TAL-PrA and resolved by SDS–PAGE (Figure 2A). The similar Coomassie-stained protein pattern for the TAL-PrA and wild-type control samples in Figure 2A demonstrates that the co-enrichment of contaminating proteins was a major issue for these types of approaches. Accordingly, the label-free spectral counting approach described in the Experimental Procedures section was used to identify proteins specifically enriched with the TAL-PrA. High-resolution mass spectrometry coupled with label-free proteomics was used to identify proteins and histone PTMs specifically enriched with the *GAL1* promoter chromatin (Figure 2B and Supplementary Table S1). We focused our analysis on the top 10% of enriched proteins (54 proteins) that each showed >15-fold enrichment with the TAL-PrA (Supplementary Table S1). Four of these 54 proteins (Rpb1, Rpb2, Spt16 and Gal3) are involved with active transcription of *GAL1*, and these are the same four proteins previously identified at the promoter of *GAL1* with the ChAP-MS approach (13). Rpb1 and Rpb2 are RNA polymerase components, and Spt16 is a subunit of yFACT that aids in re-organizing chromatin for RNA polymerase activity. Gal3 has previously been shown to inhibit the repressive activity of Gal80 at *GAL1* locus (25). Rpb1, Rpb2, Spt16 and Gal3 were confirmed to be associated adjacent to the TAL-PrA genomic binding site with standard ChIP (Figure 2C). Thus, the TAL-ChAP-MS approach identified precisely the same proteins as the published ChAP-MS approach during transcriptional activation at the promoter of *GAL1*, thereby validating the TAL-ChAP-MS approach for studying the local proteome of small chromatin regions and the use of label-free proteomic approaches for quantifying such enrichments. Many of the other 50 proteins identified as >15-fold enriched with TAL-PrA are typical non-specific protein associations found in affinity purifications (e.g. highly abundant metabolic and ribosomal proteins).

In addition to protein associations with the *GAL1* promoter, the following single histone PTMs were identified under transcriptionally active conditions: H3K14ac, H3K56ac, H3K79me1/me2/me3, H2BK17ac and H2AK7ac; and the following combinatorial histone PTMs: H3K9acK14ac, H3K18acK23ac, H2BK6acK11ac and H2BK11acK17ac (Figure 2B and Supplementary Table S2). The presence of H3K14ac was confirmed by standard ChIP (Figure 2C). Previously using ChAP-MS and routine ChIPs (13), a similar profile of singly acetylated H3 lysine residues was identified at the *GAL1* promoter region, thus confirming the utility of the TAL-ChAP-MS approach. In addition to the acetylations observed in the ChAP-MS study, the TAL-ChAP-MS approach additionally identified methylation of H3K79. As previously reported for the ChAP-MS approach, the TAL-ChAP-MS approach uncovered combinatorial sets (i.e. multiple PTMs on single peptides) of histone PTMs under transcriptionally active conditions at the promoter of *GAL1*. The use of a technology like TAL-ChAP-MS to identify previously unknown combinatorial modifications is crucial to understand the epigenome, as specific antibodies to combinatorial histone PTMs are not usually available for standard approaches like ChIP. In general terms, acetylation of histone lysine residues and methylation of H3K79 correlate to transcriptional activation (26); thus, the histone PTMs uncovered by TAL-ChAP-MS correlate to the active transcription state of *GAL1* in the presence of galactose.

## DISCUSSION

We describe an approach called TAL-ChAP-MS that provides for the biochemical isolation of 1-kb native chromatin sections for proteomic identification of specifically associated proteins and combinatorial histone PTMs. The described TAL-ChAP-MS approach overcomes limitations of the ChAP-MS approach (13), as genomic engineering is not necessary for TAL-based affinity enrichment and because protein enrichment with a given locus can now be determined with label-free proteomics. Even without genomic engineering of the DNA, the ChAP-MS approach does require targeting of a DNA-binding affinity enrichment reagent (i.e. the TAL protein), which has the potential to perturb the chromatin state. However, the data in Figure 1D demonstrate that transcription of *GAL1* is not altered on TAL targeting, which supports maintenance of the chromatin integrity for the studies reported here. Targeting TALs to different sequences in adjacent genomic regions that would provide for purification of overlapping chromatin sections is one possible way for investigators to overcome concerns of TAL binding (i.e. a tiling approach). The implications of the TAL-ChAP-MS approach are far-reaching as investigators can now begin to elucidate the dynamics of chromatin regulation in a site-specific and comprehensive manner. Researchers will now only need to ‘reprogram’ the DNA-binding specificity of the TAL protein to obtain a unique affinity purification reagent for their chromosome region of interest. Using the TAL-ChAP-MS approach brings researchers closer to being able to take molecular ‘snapshots’ of the assembly and disassembly of proteins on chromatin and how epigenetic states are modulated at small genomic loci.

## SUPPLEMENTARY DATA

Supplemental Data are available at NAR Online.

## FUNDING

National Institutes of Health grants (NIH) [R01GM106024], [P30GM103450], [P20GM103429] and [UL1TR000039]. Funding for open access charge: NIH [R01GM106024 to A.J.T. and S.D.T.].

*Conflict of interest statement*. None declared.

## Supplementary Material

Supplementary Data
