# Supplementary material for: Purification of a specific native genomic locus for proteomic analysis
Source: Nucleic Acids Res. 2013 Sep 11;41(20):e195. doi: 10.1093/nar/gkt822 (PMC3814360; doi:10.1093/nar/gkt822)
Supplement: Supplementary Data [file supp_41_20_e195__index.html]

Purification of a specific native genomic locus for proteomic analysis — Purification of a specific native genomic locus for proteomic analysis — Supplementary Data 

# Purification of a specific native genomic locus for proteomic analysis

## Supplementary Data

files

**Files in this Data Supplement:**

- Supplementary Data - xlsx file
- Supplementary Data - xlsx file
- Supplementary Data - xlsx file
